# Adjuvant whole abdominal intensity modulated radiotherapy (IMRT) for high risk stage FIGO III patients with ovarian cancer (OVAR-IMRT-01) – Pilot trial of a phase I/II study: study protocol

**DOI:** 10.1186/1471-2407-7-227

**Published:** 2007-12-19

**Authors:** Nathalie Rochet, Alexandra D Jensen, Florian Sterzing, Marc W Munter, Michael H Eichbaum, Andreas Schneeweiss, Christof Sohn, Juergen Debus, Wolfgang Harms

**Affiliations:** 1 Department of Radiation Oncology, University of Heidelberg, Im Neuenheimer Feld 400, 69120 Heidelberg, Germany; 2 Department of Radiation Oncology, German Cancer Research Center (dkfz), Im Neuenheimer Feld 280, 69120 Heidelberg, Germany; 3 Department of Gynaecology and Obstetrics, University of Heidelberg, Voßstr. 9, 69115 Heidelberg, Germany

## Abstract

**Background:**

The prognosis for patients with advanced epithelial ovarian cancer remains poor despite aggressive surgical resection and platinum-based chemotherapy. More than 60% of patients will develop recurrent disease, principally intraperitoneal, and die within 5 years. The use of whole abdominal irradiation (WAI) as consolidation therapy would appear to be a logical strategy given its ability to sterilize small tumour volumes. Despite the clinically proven efficacy of whole abdominal irradiation, the use of radiotherapy in ovarian cancer has profoundly decreased mainly due to high treatment-related toxicity. Modern intensity-modulated radiation therapy (IMRT) could allow to spare kidneys, liver, and bone marrow while still adequately covering the peritoneal cavity with a homogenous dose.

**Methods/Design:**

The OVAR-IMRT-01 study is a single center pilot trial of a phase I/II study. Patients with advanced ovarian cancer stage FIGO III (R1 or R2< 1 cm) after surgical resection and platinum-based chemotherapy will be treated with whole abdomen irradiation as consolidation therapy using intensity modulated radiation therapy (IMRT) to a total dose of 30 Gy in 1.5 Gy fractions. A total of 8 patients will be included in this trial. For treatment planning bone marrow, kidneys, liver, spinal cord, vertebral bodies and pelvic bones are defined as organs at risk. The planning target volume includes the entire peritoneal cavity plus pelvic and para-aortic node regions.

**Discussion:**

The primary endpoint of the study is the evaluation of the feasibility of intensity-modulated WAI and the evaluation of the study protocol. Secondary endpoint is evaluation of the toxicity of intensity modulated WAI before continuing with the phase I/II study. The aim is to explore the potential of IMRT as a new method for WAI to decrease the dose to kidneys, liver, bone marrow while covering the peritoneal cavity with a homogenous dose, and to implement whole abdominal intensity-modulated radiotherapy into the adjuvant multimodal treatment concept of advanced ovarian cancer FIGO stage III.

## Background

Ovarian cancer remains the number one gynaecological killer in the western world [1]. Symptoms tend to be vague; hence ovarian cancer is often detected late [2,3]. Approximately 70% of ovarian cancer patients present with advanced stage disease after it has spread beyond the genital organs and into the peritoneal cavity (stage FIGO III) or even further (stage FIGO IV, distant metastases). With a 5-year survival about 70% of patients with ovarian cancer stage II, the overall survival dramatically decreases in the advanced stages of disease, in stage III and IV it is only about 31% and 13% respectively [4,5].

The standard management of ovarian carcinoma is aggressive surgical resection (including at least total abdominal hysterectomy, bilateral adnexectomy, omentectomy, debulking of tumour masses) followed by platinum-based adjuvant chemotherapy. Generally this includes 6 courses of carboplatin or cisplatin (AUC 5) IV and paclitaxel (175 mg/m2) IV, every 3 weeks [6-11]. While the majority of patients respond to treatment, most will relapse, principally intraperitoneal, resulting in 5-year survival rates for advanced disease of approximately 20–25% [12,13].

However, subgroups of patients profiting from an even more aggressive treatment can be identified according to prognostic criteria such as stage, residual disease and grading. Patients with FIGO stage III G2/G3 with residual disease < 2 cm and FIGO stage II G3 R<2 cm are classified as high risk, with a risk of recurrence of around 80% with a 5-year survival of 25 % [14]. Hence, this subgroup of patients needs further efforts to improve outcome.

By now, several sometimes randomized trials have evaluated strategies of consolidation therapy for high risk patients (continuation of chemotherapy, other chemotherapy, high-dose chemotherapy followed by stem cell transplant, intraperitoneal chemotherapy, unspecific immunotherapy) but were not able to improve outcome significantly so far [5,9,10,15-17].

The use of whole abdominal irradiation (WAI) as a consolidation therapy would appear to be a logical strategy. The fact that whole abdominal radiotherapy can be curative in certain groups of patients has been shown by intensive studies by Dembo et al [18,19]. During the 1950s surgery and radiotherapy were the predominant treatment modalities in ovarian cancer. However, with the introduction of chemotherapy during the late 1950s and its proven effects in advanced ovarian cancer (yielding the best primary response rate), radiotherapy has been more and more abandoned mostly due to marked side effects [20]. Typically, a dose of 30 Gy is given to the whole abdomen with an anterior-posterior/posterior-anterior beam arrangement and posterior shielding of the kidneys [21]. Due to the extensive field sizes (40 cm × 30 cm or larger), a high percentage of patients experienced severe side effects, myelosuppression being the most commonly reported. Moreover, the shielding of organs at risk delivers inadequate low doses in parts of the target volume thus reducing efficiency of radiation [22-25].

Postoperative radiotherapy as a consolidation treatment with or without prior chemotherapy been investigated on numerous occasions, though initially with inconsistent and often disappointing results [26,27]. Thomas reviewed whole-abdominal pelvic irradiation in 28 published trials prior to 1992 [28,29]. Even though 700 patients were treated overall in these trials, it was impossible to reach a definitive conclusion as to the role of whole-abdominal irradiation for consolidation because of the diversity of criteria for patient selection, and the variability in the chemotherapy, surgery and radiation technique. Thomas gave some possible causes for these disappointing results in order to guide the design for future studies, the first being inappropriate patient selection based on large-volume residuum. Some authors suggest the initial residuum prior to chemotherapy should be less than 2 cm [26]. Most authors believe that radiotherapy should follow surgery and chemotherapy in case of small residual tumours (< 5 mm) or only microscopically detectable rests [30].

In addition to inappropriate patient selection, another possible explanation for the treatment failure observed in these trials is the high-degree of toxicity resulting in delayed or incomplete radiotherapy. Cumulative bone marrow toxicity is also increased in patients receiving additional chemotherapy. Inability to complete sequential therapy as planned in a significant proportion of patients may be a contributing factor to the lack of overall benefit [20,25,29]. Thus, if sequential multimodality therapy is to be tested, it is important to keep the risk of complications to a minimum maximising the probability of completing radiotherapy successfully. Severe late intestinal radiation reactions were recorded in about 10% [28]. These were associated with additional pelvic boost and previous second-look laparotomy, which is nowadays obsolete.

Recent studies have shown more promising findings. 1999 a retrospective case control study compared the outcome of patients with advanced ovarian cancer after surgery followed by chemotherapy with the combination of surgery, chemotherapy and irradiation. The results showed a significantly improved disease-free survival for patients in the radiotherapy group, and suggested that the role of radiotherapy should be re-evaluated in a prospective randomized study [30].

Also in 1999, results from 64 patients with advanced ovarian cancer without residual disease after surgery and carboplatin-based chemotherapy were randomized to either chemotherapy alone or chemotherapy plus consolidation radiotherapy. There was a significantly prolonged relapse-free interval and the overall survival in the radiotherapy arm in this study with differences being more pronounced in patients with FIGO stage III [31].

2003 a randomized trial compared whole abdominal radiation therapy, chemotherapy and no further treatment in advanced ovarian cancer (FIGO III). One hundred seventy-two patients achieved a complete surgical remission after induction chemotherapy. In the subgroup with complete pathologic remission, progression-free survival was significantly better in patients treated with radiotherapy (56% at 5 years) than with chemotherapy alone (36% at 5 years), and the untreated control group (35% at 5 years). Overall survival was also most favourable in the radiotherapy group (69% at 5 years), however, this difference failed to achieve statistical significance (P = 0,084). The number of recurrences was also lowest in the radiotherapy group. In the subgroup with microscopic residual disease, there was no significant difference in survival between the radiotherapy and chemotherapy group [32].

One trial published in 2005 aimed to determine the outcome associated with the use of whole abdominal radiotherapy in women with ovarian cancer, even after positive second-look laparotomy (SLL) or second look debulking for recurrent disease. The investigators came to the conclusion that radiotherapy appeared to be an efficacious treatment option for patients with microscopic disease at SLL, but does not appear to be more effective than chemotherapy in patients with macroscopic disease at SLL [33].

2003 a swedish systematic review of radiation therapy trials came to the conclusion there might be some evidence to suggest that radiotherapy plays a role as consolidation therapy in patients with advanced ovarian cancer and pathologically complete response after chemotherapy [34].

In summary, the review of available literature demonstrates a rationale in favour of consolidation whole abdominal irradiation in advanced ovarian cancer. Whole abdominal irradiation after surgical resection and platinum-based adjuvant chemotherapy seems to increase disease-free and overall survival in patients with ovarian cancer disease stage FIGO III with no or only minimal residual disease. However, classical WAI technique after surgery and chemotherapy is associated with high toxicities, particularly haematological toxicity, which leads to treatment interruptions.

Technical developments in radiotherapy techniques like intensity-modulated radiation therapy (IMRT) have now the potential to spare organs at risk (OARs) as kidneys, liver, and bone marrow [35,36]. Hence, these techniques might significantly decrease the toxicity while still adequately covering the peritoneal cavity with a homogenous dose [37]. Intensity-modulated radiation therapy (IMRT) is a relatively new but increasingly accepted technology delivering radiation more precisely to the tumour while sparing surrounding normal tissue [18,19,21,22,38-40]. Treatment planning and delivery with IMRT are a standard treatment technique now used for sites such as prostate, paraspinal tumours, tumours of the brain, and head&neck tumours. This technique is also increasingly explored for more challenging sites like gastro-intestinal tumours or breast cancer [35,41-48]. The challenge of this study is the application of this technique to a large and complexly shaped target volume like the peritoneal cavity [49,50].

Previous work by our group included inversely planned IMRT-treatments as a plan comparison based on CT-scans obtained from an abdominal staging CT scan of a patient with advanced ovarian cancer after receiving her informed consent. It could be shown that, in principle, IMRT of the whole peritoneal cavity is possible, though patient immobilisation using a vacuum mattress and a scotch cast mask was needed to obtain the required accuracy necessary in body stereotaxy.

## Methods/Design

### Study design

The study is designed as a single center pilot trial of a phase I/II study evaluating the feasibility and toxicity of intensity-modulated WAI.

### Study objectives

The primary objective is to evaluate the feasibility of planning and treatment application of intensity modulated whole abdominal irradiation and to evaluate the study protocol. Secondary endpoint is evaluation of the toxicity in intensity-modulated whole abdominal irradiation before the initiation of a prospective phase I/II study.

### Trial organisation

OVAR-IMRT-01 has been designed by the study initiators at the Department of Radiation Oncology at the University of Heidelberg. The trial is carried out by the Department of Radiation Oncology at the University of Heidelberg in co-operation with the German Cancer Research Center (dkfz) and Department of Gynaecology and Obstetrics.

### Coordination

The overall coordination is performed by the Department of Radiation Oncology at the University of Heidelberg. This department is also responsible for the overall trial management, database management, quality assurance including monitoring and reporting.

### Investigators

The study investigators are experienced radiation oncologists specialized in the treatment of patients with gynaecological malignancies. Patients will be recruited and treated by the physicians of the Department of Radiation Oncology of the University of Heidelberg.

### Ethics, informed consent and safety

The final protocol was approved by the ethics committee of the University of Heidelberg, Heidelberg, Germany (Nr: 176/2005) and by an independent expert group of the German Society for Radio-oncology (DEGRO). This study complies with the Helsinki Declaration in its recent German version, the Medical Association code of conduct, the principles of Good Clinical Practice (GCP) and the Federal Data Protection Act. The trial will also be carried out in keeping with local legal and regulatory requirements. The medical secrecy and the Federal Data Protection Act will be followed. The ClinicalTrials.gov Protocol ID is NCT00527631.

### Patient selection

Inclusion criteria into the study protocol are:

- histologically confirmed ovarian cancer stage FIGO III

- grade 2 or 3

- maximal typical surgical resection (including at least total abdominal hysterectomy, bilateral adnexectomy, omentectomy, debulking of tumour masses)

- postoperative residual tumour of less than 1 cm (maximal diameter of largest tumour residual is 1 cm)

- adjuvant chemotherapy consisting of six courses of carboplatin/paclitaxel or carboplatin/docetaxel

- mplete remission after chemotherapy

- Karnofsky performance Score >60

- patients > 18 and < 75 years of age

- written informed consent

### Exclusion criteria

- stage FIGO I or II

- stage IV (distal metastasis) stage

- III R2 > 1 cm

- delayed wound healing post laparotomy

- obesity

- neutrophil count (ANC) < 2000/ml before radiotherapy

- platelets < 100000/ml

- connective tissue disease, sclerodermia

- clinically active renal, hepatic, cardiac, metabolic, respiratory, coagulation or haematopoietic disease

- participation in another clinical trial

- patient refusal

### Statistical calculations for trial sample size

The primary objective is to evaluate the feasibility of planning and application of intensity-modulated whole abdominal irradiation. 8 patients will be included into the study. If planning and treatment application is not feasible for the first 5 patients the study will be discontinued.

### Adverse events

Radiotherapy-related toxicities will be assessed using the NCI Common Toxicity Criteria (CTC). Toxicity will be evaluated pre-treatment, weekly during radiation therapy (blood count, electrolytes, chemistry, clinical examination, patient visits) and at follow-up. Unacceptable toxicity is defined as unpredictable or irreversible grade 4 toxicity.

Expectable possible acute toxicities (up to 3 months post radiation therapy) are fatigue, loss of appetite, weight loss, skin toxicity (epitheliolysis, erythema, hyperpigmentation), nausea, vomiting, irritable bowel syndrome, diarrhea, intestinal bleeding, proctitis, dysuria, interstitial nephritis, hepatitis, pneumonitis, haematological toxicity with thrombocytopenia, leukocytopenia, anaemia. These symptoms can be medically treated and usually resolve within 2–3 weeks. However, transient parenteral nutrition and hydratation might be necessary in some cases. All acute toxicities should completely resolve within a few weeks after radiation therapy.

Late side effects are rare and are defined as symptoms appearing at least 3 months post radiation. These could include chronic diarrhea, malabsorbtive syndrome, chronic bladder inflammation, enterecolitis, strictures, fibroses, ulcers, chronic bleeding. Very rare symptoms are fistulation, perforation, peritonitis, intestinal necrosis, ileus necessitating surgical intervention resulting in intestinal resection/anus praeter formation. In literature reviews, severe late intestinal radiation reactions were recorded in about 10%, and were associated with pelvic boost and previous second-look laparotomy. However, patients with these risk factors are not included in this trial. Cardiac, pulmonary, hepatic and renal toxicity with accompanying renal dysfunction, teleangiectases, and spinal cord toxicities are unlikely with the dose and fractionation applied in the trial.

### Radiation therapy

Irradiation is applied as intensity-modulated radiation therapy (IMRT) using a 6 MeV linear accelerator (Siemens) with a motoric multi-leaf collimator in a step and shoot technique.

### Investigation schedule

#### Indication

The oncological treatment concept for each patient is based on interdisciplinary assessment following approved standard therapies and guidelines. Consent of the colleagues from gynaecology, internal medicine and radiation oncology are obligatory. The adjuvant treatment is defined after surgery and complete staging including abdominal and transvaginal ultrasound, chest X-ray, mammography and pelvic-abdominal CT scan if necessary, as well as pre- and post-op tumour marker (CA 125). This allows for histologically confirmed diagnosis, classification, and staging. If the patient meets the inclusion criteria chemotherapy is determined and subsequently indication for irradiation is approved.

#### Pre-therapeutic examinations

According to the interdisciplinary guidelines for therapy of ovarian cancer all patients receive 6 cycles of chemotherapy followed by a complete staging including pelvic examination, transvaginal ultrasound, and tumour markers allowing for assessment of success of the primary therapy. Additionally, abdominal and pelvic CT-scans using intravenous contrast agent are carried out. Further pre-irradiation investigations include ECG, blood count, electrolytes, coagulation, as well as blood chemistry.

#### IMRT treatment-planning

In order to ensure accurate reproducibility of patient positioning, the patient is immobilized using vacuum mattress and scotch cast head mask (arms over head). CT-scans are obtained in the immobilisation device and stereotactic body frame in 3 mm-slices. This is done in cooperation with the DKFZ according to national DIN norm and international recommendations (ICRU 50 Report 1999). Target volumes and organs at risk are defined using the appropriate in-house planning software VIRTUOS v.4.4.9 for contouring and dose calculation; MRC Konrad v.1.1.4 is used for optimisation of inversely planned intensity modulated RT. The target volume includes the whole peritoneal cavity extending from diaphragm to douglas cavity. In addition, retroperitoneal, para-aortic and pelvic nodal areas are included. Kidneys, liver, lungs, heart and spinal cord, vertebrae, femora, and pelvic bones are defined as organs at risk (OARs). A dose of 30.0 Gy is prescribed to median of GTV. No strict constraints are applied for optimization. In the context of this pilot feasibility trial the goal of optimization is a dose distribution in the PTV as homogeneous as possible and maximal sparing of organs at risk (OARs) with a high priority on liver, kidney and bones. Tolerated maximum doses to OARs must not exceed the TD5/5 for each organ. Time span allotted for treatment planning is approximately 5 days.

#### IMRT-treatment

After validation of the treatment plan by the radiation oncologist and the physicist in charge, treatment is applied in daily fractions of 5 × 1.5 Gy per week to a total dose of 30 Gy. Total treatment duration hence is 4 weeks. Isocenter and patient positioning are checked on the first fraction and then weekly with the in-room CT (Siemens Primatom). Treatment times are around 35–45 min. Treatment will be carried out on an out-patient basis unless the patient's condition requires hospital admission.

### Follow up

Patients are included into standard gynaecology follow-up program. This includes follow-up visits at 6 weeks, 3 months, 6 months, 9 months, 12 months, post treatment, and then every 6 months for 5 years. Each visit includes pelvic examination, transvaginal ultrasound and tumour marker checks. In addition, pelvic-abdominal CT-scans are performed 6, 12, 24, 36 months post treatment.

### Monitoring

Monitoring is performed according good clinical practice (GCP) guidelines. The data management will be performed by the Clinical Trial Center of the Department of Radiation Oncology, University of Heidelberg.

## Discussion

Ovarian cancer remains the number one gynaecological killer in the western world. Most ovarian cancer patients present with advanced-stage disease and are treated with cytoreductive surgery followed by platinum-based combination chemotherapy. While the majority of patients respond to treatment, most will relapse, principally intraperitoneal, such as the 5-year survival rate for advanced disease is approximately 20–25%.

The use of whole abdominal irradiation (WAI) as consolidation therapy would appear to be a logical strategy given its ability to sterilize small tumour volumes. During the 1950s surgery and radiotherapy were the predominant treatment modalities in ovarian cancer. But despite whole abdominal irradiation's (WAI) clinically proven efficacy and with the introduction of chemotherapy the use of radiotherapy in ovarian cancer has profoundly decreased mainly due to high toxicity. Technical developments in radiotherapy techniques like intensity-modulated radiation therapy (IMRT) have now the potential to spare organs at risk (OARs) as kidneys, liver, and bone marrow. Hence, these techniques might significantly decrease the toxicity while still adequately covering the peritoneal cavity with a homogenous dose.

The OVAR-IMRT-01 study is a single center pilot trial of a phase I/II study. After surgical resection and platinum-based chemotherapy patients with advanced ovarian cancer stage FIGO III (R1 or R2< 1 cm) will be treated as consolidation therapy with whole abdomen irradiation (WAI) using intensity modulated radiation therapy (IMRT) to a total dose of 30 Gy in 1.5 Gy fractions. The primary endpoint of the study is the evaluation of the feasibility of intensity modulated WAI and study protocol. Secondary endpoint is evaluation of the toxicity of intensity modulated WAI before initiating a prospective phase I/II study. The aim is to explore the potential of IMRT as a new method for WAI to lower the dose to kidneys, liver, bone marrow while covering the peritoneal cavity with a homogenous dose, and to implement whole abdominal intensity modulated radiotherapy into the adjuvant multimodal treatment concept of advanced ovarian cancer FIGO stage III.

## Abbreviations

CTC Common Toxicity Criteria

dkfz German Cancer Research Center

FIGO International Federation of Gynaecology and Obstetrics

GCP Good Clinical Practice

IMRT Intensity Modulated Radiation Therapy

PTV Planning Target Volume

SLL Second-Look Laparotomy

TD5/5 Tolerance Dose 5% over 5 years

WAI Whole Abdominal Irradiation

## Competing interests

The author(s) declare that they have no competing interests.

## Authors' contributions

NR, JD and WH planned, organized and conduct the study. ME, AS and CS recruit the patients for the study. NR, FS, MM and AJ perform planning and radiation therapy. Medical care and follow up is provided by NR and WH. All authors have read and approved the final manuscript.

## Pre-publication history

The pre-publication history for this paper can be accessed here:



## References

[B1] Heintz AP, Odicino F, Maisonneuve P, Beller U, Benedet JL, Creasman WT (2003). Carcinoma of the ovary. Int J Gynaecol Obstet.

[B2] Bast RC, Brewer M, Zou C, Hernandez MA, Daley M, Ozols R (2007). Prevention and early detection of ovarian cancer: mission impossible?. Recent Results Cancer Res.

[B3] Daly MB, Ozols RF (2004). Symptoms of ovarian cancer – where to set the bar?. JAMA.

[B4] Poveda A (2000). Advanced ovarian cancer: update, remarks and conclusions on optimal therapy. Int J Gynecol Cancer.

[B5] Stuart G, Avall-Lundqvist E, du BA, Bookman M, Bowtell D, Brady M (2005). 3rd International Ovarian Cancer Consensus Conference: outstanding issues for future consideration. Ann Oncol.

[B6] du BA, Quinn M, Thigpen T, Vermorken J, Avall-Lundqvist E, Bookman M (2005). 2004 consensus statements on the management of ovarian cancer: final document of the 3rd International Gynecologic Cancer Intergroup Ovarian Cancer Consensus Conference (GCIG OCCC 2004). Ann Oncol.

[B7] Morgan RJ, Alvarez RD, Armstrong DK, Chen LM, Copeland L, Fowler J (2006). Ovarian cancer. Clinical practice guidelines in oncology. J Natl Compr Canc Netw.

[B8] Ozols RF (2004). NICE guidelines for ovarian cancer: recommendations versus standard care. Cancer Invest.

[B9] Ozols RF (2004). Update on Gynecologic Oncology Group (GOG) trials in ovarian cancer. Cancer Invest.

[B10] Ozols RF (2006). Systemic therapy for ovarian cancer: current status and new treatments. Semin Oncol.

[B11] Ozols RF (1995). Carboplatin and paclitaxel in ovarian cancer. Semin Oncol.

[B12] Ozols RF (2005). Treatment goals in ovarian cancer. Int J Gynecol Cancer.

[B13] Heintz AP, Odicino F, Maisonneuve P, Quinn MA, Benedet JL, Creasman WT (2006). Carcinoma of the ovary. FIGO 6th Annual Report on the Results of Treatment in Gynecological Cancer. Int J Gynaecol Obstet.

[B14] Dembo AJ, Bush RS (1982). Current concepts in cancer: ovary – treatment of stages III and IV. Choice of postoperative therapy based on prognostic factors. Int J Radiat Oncol Biol Phys.

[B15] Alberts DS, Markman M, Muggia F, Ozols RF, Eldermire E, Bookman MA (2006). Proceedings of a GOG workshop on intraperitoneal therapy for ovarian cancer. Gynecol Oncol.

[B16] Ozols RF, Bookman MA, Young RC (2006). Intraperitoneal chemotherapy for ovarian cancer. N Engl J Med.

[B17] Poveda A (2003). Ovarian cancer treatment: what is new. Int J Gynecol Cancer.

[B18] Dembo AJ (1985). Abdominopelvic radiotherapy in ovarian cancer. A 10-year experience. Cancer.

[B19] Dembo AJ (1992). Epithelial ovarian cancer: the role of radiotherapy. Int J Radiat Oncol Biol Phys.

[B20] Lindner H, Willich H, Atzinger A (1990). Primary adjuvant whole abdominal irradiation in ovarian carcinoma. Int J Radiat Oncol Biol Phys.

[B21] Cardenes H, Randall ME (2000). Radiotherapy in epithelial ovarian cancer: state of the art. Forum (Genova).

[B22] Fyles AW, Dembo AJ, Bush RS, Levin W, Manchul LA, Pringle JF (1992). Analysis of complications in patients treated with abdomino-pelvic radiation therapy for ovarian carcinoma. Int J Radiat Oncol Biol Phys.

[B23] LaRouere J, Perez-Tamayo C, Fraass B, Tesser R, Lichter AS, Roberts J (1989). Optimal coverage of peritoneal surface in whole abdominal radiation for ovarian neoplasms. Int J Radiat Oncol Biol Phys.

[B24] Reinfuss M, Kojs Z, Skolyszewski J (1993). External beam radiotherapy in the management of ovarian carcinoma. Radiother Oncol.

[B25] Whelan TJ, Dembo AJ, Bush RS, Sturgeon JF, Fine S, Pringle JF (1992). Complications of whole abdominal and pelvic radiotherapy following chemotherapy for advanced ovarian cancer. Int J Radiat Oncol Biol Phys.

[B26] Lawton F, Luesley D, Blackledge G, Hilton C, Kelly K, Latief T (1990). A randomized trial comparing whole abdominal radiotherapy with chemotherapy following cisplatinum cytoreduction in epithelial ovarian cancer. West Midlands Ovarian Cancer Group Trial II. Clin Oncol (R Coll Radiol).

[B27] Ledermann JA, Dembo AJ, Sturgeon JF, Fine S, Bush RS, Fyles AW (1991). Outcome of patients with unfavorable optimally cytoreduced ovarian cancer treated with chemotherapy and whole abdominal radiation. Gynecol Oncol.

[B28] Thomas GM (1993). Is there a role for consolidation or salvage radiotherapy after chemotherapy in advanced epithelial ovarian cancer?. Gynecol Oncol.

[B29] Thomas GM, Dembo AJ (1993). Integrating radiation therapy into the management of ovarian cancer. Cancer.

[B30] Einhorn N, Lundell M, Nilsson B, Ragnarsson-Olding B, Sjovall K (1999). Is there place for radiotherapy in the treatment of advanced ovarian cancer?. Radiother Oncol.

[B31] Pickel H, Lahousen M, Petru E, Stettner H, Hackl A, Kapp K (1999). Consolidation radiotherapy after carboplatin-based chemotherapy in radically operated advanced ovarian cancer. Gynecol Oncol.

[B32] Sorbe B (2003). Consolidation treatment of advanced (FIGO stage III) ovarian carcinoma in complete surgical remission after induction chemotherapy: a randomized, controlled, clinical trial comparing whole abdominal radiotherapy, chemotherapy, and no further treatment. Int J Gynecol Cancer.

[B33] Dowdy SC, Metzinger DS, Gebhart JB, Srivatsa P, Haddock MG, Suman VJ (2005). Salvage whole-abdominal radiation therapy after second-look laparotomy or secondary debulking surgery in patients with ovarian cancer. Gynecol Oncol.

[B34] Einhorn N, Trope C, Ridderheim M, Boman K, Sorbe B, Cavallin-Stahl E (2003). A systematic overview of radiation therapy effects in ovarian cancer. Acta Oncol.

[B35] Hurkmans CW, Cho BC, Damen E, Zijp L, Mijnheer BJ (2002). Reduction of cardiac and lung complication probabilities after breast irradiation using conformal radiotherapy with or without intensity modulation. Radiother Oncol.

[B36] Mundt AJ, Lujan AE, Rotmensch J, Waggoner SE, Yamada SD, Fleming G (2002). Intensity-modulated whole pelvic radiotherapy in women with gynecologic malignancies. Int J Radiat Oncol Biol Phys.

[B37] Lujan AE, Mundt AJ, Yamada SD, Rotmensch J, Roeske JC (2003). Intensity-modulated radiotherapy as a means of reducing dose to bone marrow in gynecologic patients receiving whole pelvic radiotherapy. Int J Radiat Oncol Biol Phys.

[B38] Fyles AW, Thomas GM, Pintilie M, Ackerman I, Levin W (1998). A randomized study of two doses of abdominopelvic radiation therapy for patients with optimally debulked Stage I, II, and III ovarian cancer. Int J Radiat Oncol Biol Phys.

[B39] Nutting C (2003). Intensity-modulated radiotherapy (IMRT): the most important advance in radiotherapy since the linear accelerator?. Br J Radiol.

[B40] Fiorino C, Dell'Oca I, Pierelli A, Broggi S, De Martin E, Di Muzio N (2006). Significant improvement in normal tissue sparing and target coverage for head and neck cancer by means of helical tomotherapy. Radiother Oncol.

[B41] Nutting C, Dearnaley DP, Webb S (2000). Intensity modulated radiation therapy: a clinical review. Br J Radiol.

[B42] Chao KS, Ozyigit G, Tran BN, Cengiz M, Dempsey JF, Low DA (2003). Patterns of failure in patients receiving definitive and postoperative IMRT for head-and-neck cancer. Int J Radiat Oncol Biol Phys.

[B43] Debus J, Zierhut D, Didinger B, Schlegel W, Wannenmacher M (2002). Inverse planning and intensity-modulated radiotherapy in patients with prostate cancer. Front Radiat Ther Oncol.

[B44] Didinger B, Schulz-Ertner D, Wannenmacher M, Debus J (2003). [Modern techniques in the radiotherapy of prostate cancer. Non-surgical treatment options for localized stages]. Radiologe.

[B45] Lee N, Xia P, Quivey JM, Sultanem K, Poon I, Akazawa C (2002). Intensity-modulated radiotherapy in the treatment of nasopharyngeal carcinoma: an update of the UCSF experience. Int J Radiat Oncol Biol Phys.

[B46] Munter MW, Nill S, Thilmann C, Hof H, Hoss A, Haring P (2003). Stereotactic intensity-modulated radiation therapy (IMRT) and inverse treatment planning for advanced pleural mesothelioma. Feasibility and initial results. Strahlenther Onkol.

[B47] Munter MW, Thilmann C, Hof H, Didinger B, Rhein B, Nill S (2003). Stereotactic intensity modulated radiation therapy and inverse treatment planning for tumors of the head and neck region: clinical implementation of the step and shoot approach and first clinical results. Radiother Oncol.

[B48] Thilmann C, Zabel A, Milker-Zabel S, Schlegel W, Wannenmacher M, Debus J (2003). Number and orientation of beams in inversely planned intensity-modulated radiotherapy of the female breast and the parasternal lymph nodes. Am J Clin Oncol.

[B49] Duthoy W, De Gersem W, Vergote K, Coghe M, Boterberg T, De Deene Y (2003). Whole abdominopelvic radiotherapy (WAPRT) using intensity-modulated arc therapy (IMAT): first clinical experience. Int J Radiat Oncol Biol Phys.

[B50] Hong L, Alektiar K, Chui C, LoSasso T, Hunt M, Spirou S (2002). IMRT of large fields: whole-abdomen irradiation. Int J Radiat Oncol Biol Phys.

